# Helium and methane sources and fluxes of shallow submarine hydrothermal plumes near the Tokara Islands, Southern Japan

**DOI:** 10.1038/srep34126

**Published:** 2016-09-27

**Authors:** Hsin-Yi Wen, Yuji Sano, Naoto Takahata, Yama Tomonaga, Akizumi Ishida, Kentaro Tanaka, Takanori Kagoshima, Kotaro Shirai, Jun-ichiro Ishibashi, Hisayoshi Yokose, Urumu Tsunogai, Tsanyao F. Yang

**Affiliations:** 1 Department of Geosciences, National Taiwan University, Taiwan; 2 Atmosphere and Ocean Research Institute, The University of Tokyo, Japan; 3 Department of Earth and Planetary Sciences, Kyushu University, Japan; 4 Graduate School of Science and Technology, Kumamoto University, Japan; 5 Graduate School of Environmental Studies, Nagoya University, Japan

## Abstract

Shallow submarine volcanoes have been newly discovered near the Tokara Islands, which are situated at the volcanic front of the northern Ryukyu Arc in southern Japan. Here, we report for the first time the volatile geochemistry of shallow hydrothermal plumes, which were sampled using a CTD-RMS system after analyzing water column images collected by multi-beam echo sounder surveys. These surveys were performed during the research cruise KS-14-10 of the R/V Shinsei Maru in a region stretching from the Wakamiko Crater to the Tokara Islands. The ^3^He flux and methane flux in the investigated area are estimated to be (0.99–2.6) × 10^4^ atoms/cm^2^/sec and 6–60 t/yr, respectively. The methane in the region of the Tokara Islands is a mix between abiotic methane similar to that found in the East Pacific Rise and thermogenic one. Methane at the Wakamiko Crater is of abiotic origin but affected by isotopic fractionation through rapid microbial oxidation. The helium isotopes suggest the presence of subduction-type mantle helium at the Wakamiko Crater, while a larger crustal component is found close to the Tokara Islands. This suggests that the Tokara Islands submarine volcanoes are a key feature of the transition zone between the volcanic front and the spreading back-arc basin.

Hydrothermal activity provides geochemical information from the interior of the Earth (e.g., the mantle) at its surface (e.g., the atmosphere or the ocean)^1^. Shallow hydrothermal fluids emitted from seafloor vents and/or volcanoes generally form plumes in which this information is preserved. Therefore, submarine hydrothermal plumes represent a means to observe and understand regional tectonic settings and fluid circulation. In addition, shallow volcanic eruptions pose a major threat to nearby ships and boats, as a zone of lower water density causes a loss of buoyancy and increases the risk of sinking. In 1944, during the eruption of the Kick ‘em Jenny volcano offshore of the northern coast of Grenada, a passenger vessel sank while crossing the degassing region, resulting in the deaths of 60 people^2^. The Myojinsho volcano erupted in September 1952 and caused damage to a research vessel, which killed all 31 passengers^2^,^3^ in Japan. These dramatic events highlight the necessity and importance of studying submarine volcanic activity in the form of time series.

Helium is a chemically inert noble gas, and this characteristic is an advantage in deciphering the gas source. The isotope ratio ^3^He/^4^He shows a relationship with global geotectonic settings. Helium-3, in particular, is considered the most important volatile tracer of mantle-derived materials^4^,^5^. In addition, methane plays an important role in the greenhouse effect in the atmosphere and in the chemistry of ozone reduction. Major emissions of methane into the atmosphere originate from the biosphere (e.g., wetlands, rice paddies and animals), the geosphere (e.g., hydrocarbon basins and geothermal areas) and anthropogenic activity (e.g., natural gas production and distribution and coal mining)^6^,^7^. Methane is one of the most common and abundant gas species in submarine hydrothermal fluids. The concentrations of methane in hydrothermal fluids are often 10^4^–10^7^ times higher than those in ambient ocean water^8^,^9^. The stable carbon isotopic composition of methane can be used to understand the global carbon cycle and to characterize geological and microbial consumption processes^10^,^11^. The ratios of the elemental abundance of carbon and helium (C/^3^He) together with carbon isotope ratios (δ^13^C) may provide key information on the origin of the carbon^12^. Their fluxes from the solid Earth to the atmosphere may provide useful information on the geochemical cycle and the evolution of the atmosphere and ocean^13^.

The Ryukyu Arc, a typical trench-arc system, is formed by the Philippine Sea plate subducting northwestward beneath the Eurasian plate, with variable convergent rates in the range of 4 to 7 cm/yr^14^. The Ryukyu Arc extends approximately 1,200 km from Kyushu Island (Japan) to Taiwan and can be classified into three segments, namely north, central and south Ryukyu Arc. The separations between these segments are marked by the Tokara Strait and the Kerama Gap. A new chain of submarine volcanoes has been identified by the detailed topography and petrology survey conducted during the cruise KS-14-10 of the R/V Shinsei Maru. This chain of submarine volcanoes has been classified as part of the Tokara Islands and as a part of the volcanic front of the Ryukyu Arc^15^ (Fig. 1a). To elucidate the regional tectonic setting and the formation of submarine volcanoes, we integrate geomorphological, geophysical and geochemical proxies from a field cruise survey during which we collected water samples from various hydrothermal plumes. We also report the first water column images acquired using multi-beam echo sounder techniques in the region of the Tokara Islands (Daiichi-Amami Knoll and Kotakara Shima; see Fig. 1 and Supplementary Video). These images suggest that degassing in form of bubbles occurs from the investigated shallow hydrothermal systems. Furthermore, we estimate the helium and methane fluxes and the origins of the respective gas species based on the elemental and isotopic analyses of the acquired samples. Our results may be useful for future research related to the carbon cycle and for the risk management of submarine volcanic eruptions in the investigated areas.

## Results

### Daiichi-Amami Knoll

**Figure 1:**
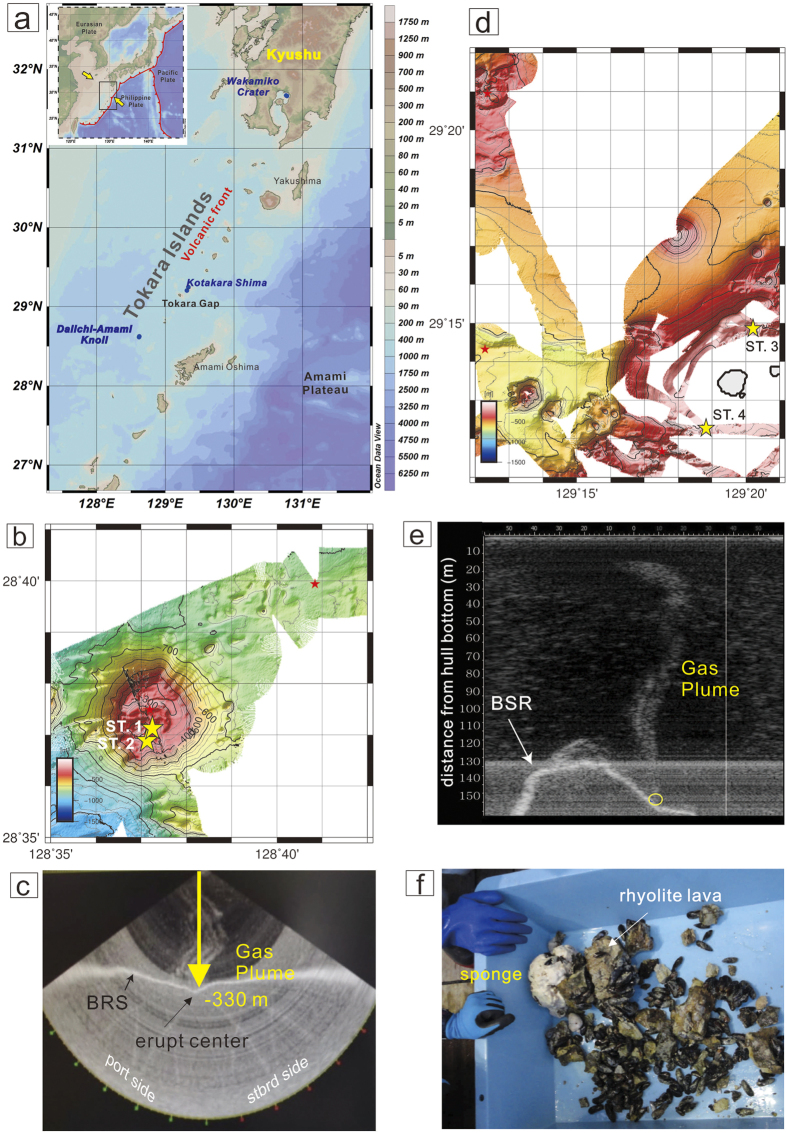
(**a**) Tectonic setting and location map of the Tokara Islands; bathymetric maps and water column images of Daiichi-Amami Knoll (**b,c**) and Kotakara Shima (**d,e**); rhyolite lava and cold seep mussels (**f**) at Daiichi-Amami Knoll. Stars on the bathymetric maps (**b** and **d**) represent the CTD operated sites during this cruise, and the yellow stars are the stations shown in Fig. 2; ST. refers to the water column sample station. The yellow circles in both (**c,e**) indicate eruptive centers; BSR means the bottom-simulating reflector. (**a**) was prepared using the Ocean Data View software^50^ and both (**b,d**) were produced with GMT version 5.3.0 (http://gmt.soest.hawaii.edu).

**Figure 2:**
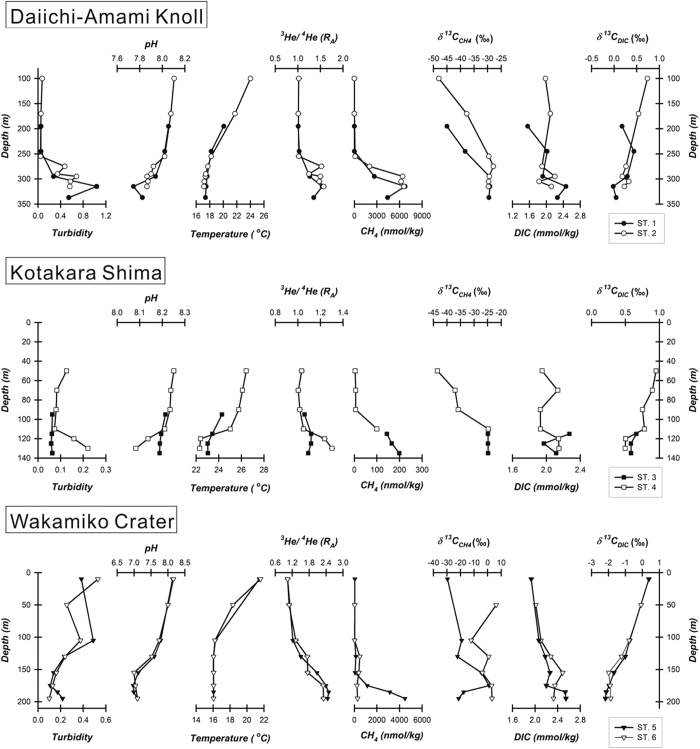
Depth profiles of turbidity, pH, temperature, helium isotope ratios, concentrations and δ^13^C values of methane, and concentration and δ^13^C of dissolved inorganic carbon (DIC) obtained at Daiichi-Amami Knoll, Kotakara Shima and Wakamiko Crater. The depth of the sampling point below the sea surface is used as the vertical axis. Black and open circles represent samples taken at station 1 and station 2, respectively, in the region of Daiichi-Amami Knoll. The results of station 3 and station 4 at Kotakara Shima are represented by black and open squares, respectively. The sampling locations of these stations at Daiichi-Amami Knoll and Kotakara Shima are shown in Fig. 1a,d, respectively. The depth profiles at the Wakamiko Crater are shown as station 5 (black inverted triangles) and station 6 (open inverted triangles).

Bathymetric mapping and dredge sampling were carried out at the Daiichi-Amami Knoll (Fig. 1b). In this region, rhyolite lava and cold seep mussels were recovered by dredging (Fig. 1f), implying that the hydrothermal system is an environment rich in methane and hydrogen sulfide. We collected hydrothermal plume samples via CTD-RMS hydrocasts immediately after the water column image analysis (Fig. 1c). The ^3^He/^4^He ratios, CH_4_ concentrations and δ^13^C_CH4_ values of the seawater samples acquired at Daiichi-Amami Knoll fall in the ranges of 1.01 to 1.57 R_a_ (where R_a_ is the atmospheric ^3^He/^4^He ratio of 1.382 × 10^−6^)^16^, 3 to 6738 nM and −48.0 to −27.9‰, respectively. Strong hydrothermal activity was identified at a water depth range between 275 to 300 m based on high turbidity, low pH values, high ^3^He/^4^He ratios and significant CH_4_ concentration anomalies corresponding to more positive δ^13^C_CH4_ values (Fig. 2), although no apparent temperature anomaly was detected.

### Kotakara Shima

Both the bathymetric map and water column imaging (Fig. 1d,e) indicate the presence of shallow submarine volcanoes at a water depth of approximately 130 m in the region of Kotakara Shima. Gas geochemistry profiles of the shallow hydrothermal and/or seepage plume are shown in Fig. 2. The ^3^He/^4^He ratios varied from 1.00 to 1.30 R_a_. The methane concentrations and carbon isotope ratios fall in the range of 4 to 199 nM and −43.5 to −24.8‰, respectively. Basic seawater physico-chemical parameters (turbidity, pH, and temperature) and gas geochemistry parameters (helium isotope ratios, methane concentrations and δ^13^C_CH4_ values) showed anomalies at the same water depth of approximately 130 m and indicate the presence of active fluid emissions at the seafloor. The observed negative anomaly in the seawater temperature profile suggests the occurrence of cold seepage enriched in mantle helium.

### Wakamiko Crater

The ^3^He/^4^He ratios increased with increasing water depth from 1.04 to 2.49 R_a_, which agrees with previous studies^17^,^18^. The methane concentration profile, following a similar pattern as the helium isotope ratio profile, increased with depth from 22 to 4478 nM. The stable carbon isotopes of methane showed large variations, covering a range of values from −29.6 to 6.3‰. Hydrothermal activity was identified at a depth of approximately 150 m based on low pH values and high ^3^He/^4^He ratios measured in the seawater samples. A significant methane anomaly was observed at a depth of approximately 200 m. A positive correlation exists between the ^3^He/^4^He ratios and the methane contents, similar to the Daiichi-Amami Knoll and Kotakara Shima samples.

## Discussion

**Figure 3:**
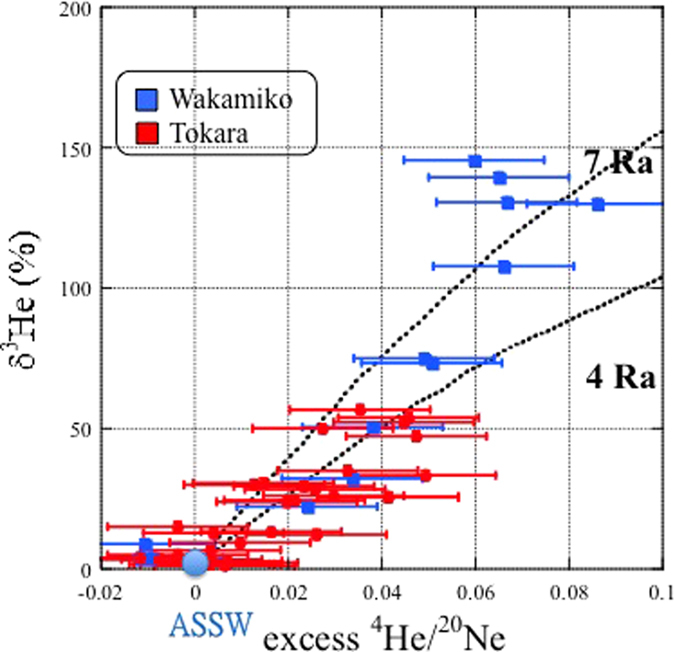
Origin of helium in a shallow marine hydrothermal system. Error assigned to the symbol is 2σ. ASSW is air-saturated seawater.

A positive relationship between the δ^3^He value (where δ^3^He = (R − 1) × 100, R = ^3^He/^4^He) and the excess ^4^He/^20^Ne ratio relative to air-saturated seawater values suggests two-component mixing between the atmospheric and volcanic sources (Fig. 3). The end member for the Wakamiko Crater samples exhibits a subduction-type mantle helium signature of approximately 7 R_a_, which is consistent gases from volcanoes and hot springs in the Circum-Pacific belt with the high ^3^He/^4^He ratios (up to 7.86 Ra)^19^. The Tokara Islands helium isotopic composition is affected by addition of a larger amount of crustal He (which has R/R_a_ typical of 0.02–0.03 R_a_) lowering the mantle value down to 4 R_a_.

The helium isotope ratios of hydrothermal fluids from different geologic settings are listed in Table 1. The helium at the frontal arc of subduction zone exhibits a crustal signature characterized by low ^3^He/^4^He ratios and biogenic methane, while at the volcanic arc region exhibits mantle helium with high ^3^He/^4^He ratios and thermogenic methane. The Tokara samples show an intermediate signature, and the tectonic implications are described later.

Helium and methane fluxes provide further geochemical information. It is possible to calculate the ^3^He flux based on the observed ^3^He concentration gradients and a simple steady-state diffusion model. Assuming that the concentration of neon dissolved in seawater is at atmospheric equilibrium (i.e., 1.86 × 10^−7^ cm^3^ STP/g H_2_O), it is possible to calculate the ^3^He gradient at each depth using the measured ^3^He/^4^He and ^4^He/^20^Ne ratios. Assuming a vertical eddy diffusivity of 100 cm^2^/s^20^, the shallow hydrothermal and/or low-temperature seepage ^3^He fluxes are estimated to be (1.9 ± 0.2) × 10^4^, (9.9 ± 4.5) × 10^3^ and (2.6 ± 0.3) × 10^4^ atoms/cm^2^/s at Daiichi-Amami Knoll, Kotakara Shima and the Wakamiko Crater, respectively. The ^3^He fluxes at the Wakamiko Crater and the Tokara Islands contribute 0.01% and 0.07% of the total ^3^He emissions (8.97 × 10^24^ atoms/yr) along the Japan arc^4^. The ^3^He flux of the Tokara Islands is four orders of magnitude higher than that of the seafloor in the southern Okinawa Trough (1.6 ^3^He atoms/cm^2^/s)^21^. The ^3^He flux of the Tokara Islands is derived from the venting of the submarine volcanoes and could be regarded as direct emissions from the crater. However, the ^3^He flux of the southern Okinawa Trough is represented as a diffusive flux from a volcanic edifice^21^. These phenomena are similar to phenomena in the study of terrestrial volcanoes, the diffuse degassing fluxes through volcanic edifice and plume degassing from summit craters might be complementary^22^. The ^3^He flux at the Wakamiko Crater is similar to a previously reported flux estimate^18^, implying that the volcanic activity was stable between 2010 and 2014.

There is no significant difference in ^3^He flux between the Tokara Islands and the Wakamiko Crater. However, the estimated end members representing the sources of terrigenic helium imply a larger crustal contribution, i.e., a larger share of ^4^He, at the Tokara Islands. As both regions are located at the volcanic front of the Ryukyu Arc and its extension, the tectonic settings likely play an important role in determining the different terrigenic He isotope contributions at the respective locations.

The slab bending beneath the north-central Ryukyu Arc, based on the information provided by the hypocentral earthquake distribution, is characterized by different dip angles to the north and south of the Tokara Strait, varying from sharp (e.g., 70° dips down to 80 km depth) in the north to gentle (40–50° dips) in the south^23^,^24^,^25^. Based on variations in geochemical parameters (e.g., Sr, Nd, and Pb isotopes) within the north Ryukyu Arc, various lavas from south Kyushu and the north-central Ryukyu arc were derived from mantle material of Indian Ocean-type and mantle wedge material of Pacific-type, respectively^25^.

In the subduction zone, the relationship between helium isotope ratios and geotectonic setting is well documented (e.g. Japan^19^ and southern Italy^26^). In the fore arc region is characterized by crustal helium; while in the volcanic arc and back arc region shows mantle helium. In light of these geophysical and geochemical insights, we propose that the Tokara Islands represent a transition zone from island arc volcanism to back-arc basin spreading volcanism with a south-north orientation; thus, the helium isotopes are somewhat low.

Submarine hydrothermal systems contain many gases. Methane is a useful species as well as an effective chemical tracer and can be used to identify the source of carbon. The positive correlation between ^3^He and methane concentrations suggests that mantle helium may be accompanied by methane. A least squares fitting of ^3^He to methane concentrations allows the determination of end members of the CH_4_/^3^He ratios of (3.5 ± 0.2) × 10^9^, (7.1 ± 0.7) × 10^8^ and (9.3 ± 6.6) × 10^8^ in the regions of Daiichi-Amami Knoll, Kotakara Shima and the Wakamiko Crater, respectively. The CH_4_/^3^He ratio at Daiichi-Amami Knoll is comparable to the Rainbow hydrothermal site on the Mid-Atlantic Ridge (1.3 × 10^8^; ref. 27). At the Wakamiko Crater, CH_4_/^3^He ratio is similar to the estimate of ~10^9^ in a previous report^17^. Additionally, the ratios of all three of these regions are close to the ratios of the Okinawa Trough, i.e., (7.6–14) × 10^8^ (refs 28 and 29). Based on the CH_4_/^3^He ratio and ^3^He flux at Daiichi-Amami Knoll, we estimate a methane flux of 6.62 × 10^13^ atoms/cm^2^/s. The methane fluxes at Kotakara Shima and the Wakamiko Crater are approximately one tenth and one third of the flux at Daiichi-Amami Knoll, respectively. Assuming a degassing area of 1 × 10^5^ m^2^ (approximately 300 m × 300 m) based on the topographic highs, the total methane emissions of the Toakara Islands and the Wakamiko Crater are approximately 66 and 22 t/yr, respectively. These observed CH_4_ fluxes are higher than the ones found in the major geological methane emission regions, mud volcanoes, gas hydrate areas and methane-rich gas in Taiwan^30^,^31^,^32^ (Supplementary Table). Thus, methane contributed from geosphere, especially by shallow submarine systems, is an important component in the global natural methane budget.

The estimated end-member δ^13^C_CH4_ value of the Tokara Islands is more positive, i.e., enriched in thermogenic methane, than the natural gases in brines from the gas fields in southwest Japan (−38.9 to −67.5‰ PDB^33^) but more negative than the gases observed in the hydrothermal fluids from mid-ocean ridge regions (−8.6 to −20‰ PDB^5^. However, the estimated end-member δ^13^C_CH4_ value at the Tokara Islands is similar to the hydrothermal fluids at Minami-Ensei and Yonaguni in the Okinawa Trough (−25 to −26.9‰ PDB^34^). Together with the carbon isotopes of methane-rich gas in Japan^35^, we conclude the methane origin related to the geological setting. The South Kanto region represents a fore-arc region of the subduction zone characterized by biogenic methane with light δ^13^C_CH4_ values and low ^3^He/^4^He ratios, while the back-arc region (Akita and Niigata) features thermogenic methane with high ^3^He/^4^He ratios. In this study, the Tokara Islands are located between the fore-arc and back-arc systems at the volcanic front; therefore, the origin of the methane is a mixture of thermogenic and biogenic methane.

The plot of δ^13^C_CH4_ versus 1/CH_4_ for the Wakamiko Crater is too scattered to identify the end member of the δ^13^C_CH4_ values. This implies that the methane of the hydrothermal plume is not affected only by the mixing of terrigenic fluids with seawater. The profiles at the Wakamiko Crater (Fig. 2) show a rapid decrease in CH_4_ concentrations, especially in the interval of 150 to 200 m at station 5, but increasing trends in the δ^13^C_CH4_ values not only at station 5 but also at station 6 within the depth interval of 50 to 100 m. These phenomena suggest additional microbial oxidation activity is present within the hydrothermal plume, as microbial oxidation processes tend to preferentially consume CH_4_ molecules with lighter carbon isotopes (^12^C) than molecules with heavier carbon isotopes (^13^C)^8^,^9^,^36^.

**Figure 4:**
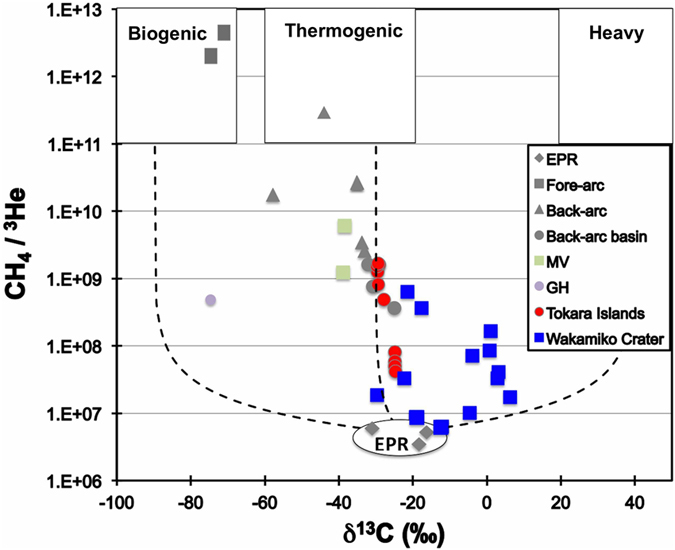
Correlation diagram between stable carbon isotopes of methane (δ^13^C) and the CH_4_/^3^He ratios in hydrothermal/cold seep plumes. Four-component mixing model for methane origin. The end-members include biogenic, thermogenic, heavy and East Pacific Rise (EPR) type. All data are from Table 1 and Supplementary Table.

Figure 4 shows the relationship between the δ^13^C_CH4_ values and CH_4_/^3^He ratios measured in the samples. There can be four major end members for the origin of methane in shallow marine hydrothermal systems: (1) abiogenic methane produced by chemical reactions, as observed on the East Pacific Rise (EPR); (2) biogenic methane produced by microbial activity utilizing inorganic carbon; (3) thermogenic methane from the thermal decomposition of organic matter; and (4) oxidized methane with heavier carbon isotope values formed through microbial fractionation in old gas plumes^9^. Most of the data from the Tokara Islands (red circles in Fig. 4) indicate mixing between EPR-type abiogenic methane and thermogenic methane, similar to the data from the Okinawa Trough^28^,^29^. In these regions, the samples closest to the sea surface are characterized by relatively negative δ^13^C_CH4_ values and lower CH_4_/^3^He ratios, implying that the end members of methane are not only affected by thermogenic methane and EPR-type methane but also by biogenic influences. The hydrothermal vents in these regions are relatively shallow, suggesting that the presence of thermogenic CH_4_ in the plumes is caused by magmatic activity, as the methane is carried from the deep lithosphere towards the seafloor by vertical hydrothermal fluid migration. However, the data from the Wakamiko Crater (blue squares) appear to be characterized by a different end-member system from the data at the Tokara Islands in Fig. 4. Previous studies suggested that the origin of the methane at Wakamiko Crater is thermogenic due to volcanic heat interacting with organic matter in sediments and high ammonium concentrations in sediment pore water^37^. The Wakamiko Crater is located within Kagoshima Bay, where organic matter is supplied by surrounding lands and the seawater is affected by the basin morphology and somewhat stagnant currents. However, as mentioned above, in the hydrothermal plumes at the Wakamiko Crater, more complex processes seem to affect the dissolved gas species in the water column. Considering the geographical settings and the results of previous studies, we conclude that the thermogenic methane carried by the emitted hydrothermal fluids and the hydrothermal plumes experiences methane oxidation, resulting in microbial fractionation and isotopically heavy methane.

In summary, we have carried out a research cruise on the R/V Shinsei Maru (expedition KS-14-10) in a region stretching from the Wakamiko Crater in Kagoshima Bay to the sea close to the Tokara Islands in SW Japan. The ^3^He and CH_4_ fluxes in the investigated shallow submarine hydrothermal systems are estimated to be (1.0–2.6) × 10^4^ atoms/cm^2^/s and 6–60 t/yr, respectively. The methane fluxes are not negligibly small compared with major geological emissions. The mantle helium contribution is smaller at the Tokara Islands than at the Wakamiko Crater, which suggests that the volcanic front exhibits a mixing pattern between fore arc and volcanic front signatures.

## Methods

### Sampling and on-site data acquisition

The multi-beam echo sounder survey can provide *in situ* preliminary submarine plume information, such as water column imaging, which allows rapid identification of the ideal location for seawater sampling. The CTD-CMS system used in this work consists of a CTD (Conductivity Temperature Depth profiler), a CMS (Carousel Multiple Sampling system), 24 Niskin bottles, and a turbidity meter that allows detection of hydrothermal plumes based on turbidity anomalies in the water column.

### Noble-gas analysis

The water in the Niskin bottles was immediately transferred to approximately 60-cm-long copper tubes without exposure to the atmosphere. Both ends of the tubes were sealed airtight by steel clamps for storage. In the laboratory, we connected the tubes to a high-vacuum line with a lead glass container to extract the dissolved gases from the seawater samples based on the displacement method. The exsolved gases were transferred from the glass bottle into a purification line where helium was purified using hot titanium-zirconium getters and charcoal traps held at liquid nitrogen temperature. The ^4^He/^20^Ne ratios were measured by an online quadrupole mass spectrometer (QMS 100, Pfeiffer). Subsequently, helium was separated from neon (and other residual gas species) using a cryogenic charcoal trap held at an extremely low temperature, 40 K^38^,^39^. The ^3^He/^4^He ratios were determined using a conventional noble gas mass spectrometer (Helix-SFT, GV Instruments) and calibrated against the Helium Standard of Japan (HESJ)^40^ at the Atmosphere and Ocean Research Institute (AORI), The University of Tokyo. The experimental errors of the ^3^He/^4^He and ^4^He/^20^Ne ratios were approximately 0.4 and 3%, respectively, at the one sigma level^41^. The measured ^3^He/^4^He ratios are reported using the R_a_-notation, where the determined He isotope ratios are normalized by the ^3^He/^4^He ratios of atmospheric air, R_a_ (i.e., 1.382 × 10^−6^ cm^3^ STP/g^16^).

### Methane concentrations and stable carbon isotope (δ^13^C) analysis

Water samples were carefully introduced from the Niskin bottles into 125 mL glass vials using a Teflon tube and avoiding the presence of air bubbles. After overflowing by twice the volume, we removed the Teflon tubes slowly and added 0.6 mL of a saturated mercury chloride solution for sterilization. The samples were capped with gray butyl rubber, sealed with an aluminum cap, and kept in a dark refrigerator until analysis. CH_4_ concentrations and δ^13^C values were simultaneously determined from one sample vial by using an isotope-ratio-monitoring gas chromatography mass spectrometer at Nagoya University. The system consisted of three parts: an extraction and purification line, a gas chromatograph (HP6850), and an isotope ratio mass spectrometer (Finnigan MAT252)^8^. The stable carbon isotopic ratios are reported using the common δ−notation in per-mil against the PDB standard^42^.

## Additional Information

**How to cite this article**: Wen, H. *et al*. Helium and methane sources and fluxes of shallow submarine hydrothermal plumes near the Tokara Islands, Southern Japan. *Sci. Rep.* **6**, 34126; doi: 10.1038/srep34126 (2016).

## Supplementary Material

Supplementary Video

Supplementary Information

## Figures and Tables

**Table 1:** He fluxes, ^3^He/^4^He ratios, CH_4_ fluxes, δ^13^C_CH4_ values and CH_4_/^3^He ratios in hydrothermal plumes and other CH_4_-rich gases.

Location	Type	^3^He flux atoms cm^−2^ s^−1^	^3^He/^4^He Ra	CH_4_ flux pmol cm^−2^ s^−1^	δ^13^C_CH4_‰	CH_4_/^3^He	Reference
EPR							
East Pacific Rise (13°N)	vent		7.6		−20 to −17	(3.1−3.9)E + 6	43
East Pacific Rise (21°N)	vent		7.8		−18 to −15	(3.4−6.5)E + 6	44,45
East Pacific Rise (27−32°S)	plume				−27 to −35	(2.0−9.7)E + 6	46
Fore-arc							
South Kanto (Narashino)	gas well		0.2			9.24E + 12	47,48
South Kanto (Yokoshiba)	gas well		0.3			2.29E + 12	47,48
South Kanto (Shirako)	gas well		0.2		−71.2	4.56E + 12	47,48
South Kanto (Chonan)	gas well		0.2			8.81E + 12	47,48
South Kanto (Heiwajima)	gas well		0.1		−74.6	2.10E + 12	47,48
Volcanic front							
Tokara Islands (Daiichi-Amami Knoll)	plume	1.90E + 04	4	110	−28 to −48	3.50E + 09	This study
Tokara Islands (Kotakara Shima)	plume	9.90E + 03	4	11	−25 to −44	7.10E + 08	This study
Kagoshima Bay (Wakamiko crater)	plume	2.60E + 04	7	40	1 to −30	9.30E + 08	This study
Back-arc							
Akita (Yoshino)	gas well		3.4		−57.8	1.73E + 10	35
Akita (Yabse)	oil well		0.9		−43.9	2.97E + 11	35,49
Niigata (M-Katagai)	gas well		3.9		−35.1	2.65E + 10	35
Niigata (Katagi)	gas well		6.2		−33.8	3.43E + 09	35
Niigata (Nakadori)	gas well		5.2		−33.1	2.58E + 09	35
Back-arc basin							
Okinawa Trough (Minami Ensei)	vent		6.99		−25	3.65E + 08	34
Okinawa Trough (Jade)	vent		6.5		−30.8	7.58E + 08	34
Okinawa Trough (Hakurei)	vent		5.81		−32.1	1.60E + 09	34
