# Supplementary material for: Helium and methane sources and fluxes of shallow submarine hydrothermal plumes near the Tokara Islands, Southern Japan
Source: Sci Rep. 2016 Sep 27;6:34126. doi: 10.1038/srep34126 (PMC5037448; doi:10.1038/srep34126)
Supplement: Supplementary Information [file srep34126-s2.doc]

**Supplementary Information**

**Helium and methane sources and fluxes of shallow submarine hydrothermal plumes near the Tokara Islands, Southern Japan**

Hsinyi Wen^1, 2, a,＊^, Yuji Sano^1, 2^, Naoto Takahata^2^, Yama Tomonaga^2, b^, Akizumi Ishida^2,c^, Kentaro Tanaka^2^, Takanori Kagoshima^2^, Kotaro Shirai^2^, Jun-Ichiro Ishibashi^3^, Hisayoshi Yokose^4^, Urumu Tsunogai^5^, Tsanyao F. Yang^§^

1. Department of Geosciences, National Taiwan University, Taiwan

2. Atmosphere and Ocean Research Institute, The University of Tokyo, Japan

3.Department of Earth and Planetary Sciences, Faculty of Science, Kyushu University, Japan

4. Graduate School of Science and Technology, Kumamoto University, Japan

5. Graduate School of Environmental Studies, Nagoya University, Japan

a. Present address: Green Energy and Environment Research Laboratories, Industrial Technology Research Institute, Taiwan

b. Present address: Eawag, Swiss Federal Institute of Aquatic Science and Technology, Dübendorf, Switzerland

c. Present address: WiscSIMS Lab, NASA Astrobiology Institute, Department of Geoscience, University of Wisconsin-Madison, USA

*Correspondence and requests for materials should be addressed to Hsinyi Wen (d99224009@ntu.edu.tw)

§. Deceased

**Supplementary Video caption**

**The video of water column images in the region of Daiichi-Amami Knoll.**

In order to investigate hydrothermal fluids at Daiichi-AmamiKnoll, Shinsei Maru navigated from the north to the south of the summit area at a speed of 8 kt. Acoustic anomaly in the WCI image is increasing gradually, indicating that its starting point is located at the crater-like depression. The plume-like acoustic anomaly ranges longer than 200 m from the crater bottom at 340 m depth.

**Supplementary Figure**

The plot of the stable carbon isotopes ratios of methane (δ^13^C_CH4_) against the reciprocal of the CH_4_ concentration (1/CH_4_) for the samples within the plumes of Daiichi-Amami Knoll and Kotakara Shima is shown in Suppl. Fig. 1. Most of the data points are located along the least-squares regression line, indicating that methane in the plumes is affected by a simple mixing process between hydrothermal fluids and ambient seawater. The intercepts of the linear regressions on the y-axis (1/CH_4_ ~0) point to the hypothetical end-member δ^13^C_CH4_ in the plumes being -29.25 ± 0.21 (1σ) and -24.38 ± 0.28 (1σ) ‰ PDB in Daiichi-Amami Knoll and Kotakara Shima, respectively. The observed values are within the range of thermogenic CH_4_ values.

**
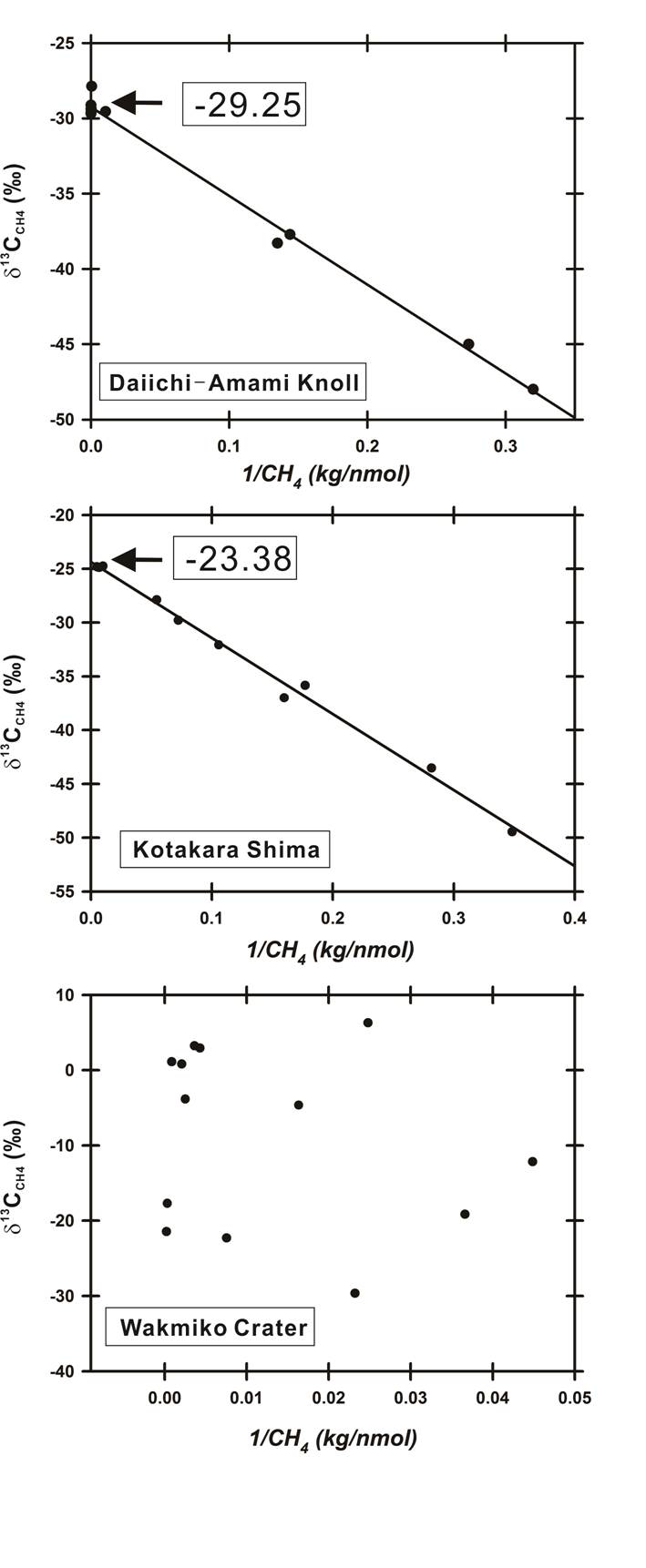
**

Figure S1. Correlation diagram between methane reciprocal concentration and carbon isotopes of the hydrothermal/coldseep plumes at Daiich-Amami Knoll, Kotakara Shima and the Wakamiko Crater.

**Supplementary Table**

**Table S1. ^3^He/^4^He ratios, CH_4_ fluxes, δ^13^C_CH4_ values and CH_4_/^3^He ratios in hydrothermal plumes and other CH_4_-rich gases.**

| Location | Type | ^3^He/^4^He | CH_4_ flux | δ^13^C_CH4_ | CH_4_/^3^He | Reference |
| --- | --- | --- | --- | --- | --- | --- |
|  |  | Ra | pmol cm^-2^ s^-1^ | ‰ |  |  |
| Tokara Islands (Daiichi-Amami Knoll) | plume | 4 | 110 | −28 to −48 | 3.50E+09 | This study |
| Tokara Islands (Ko-Takara Shima) | plume | 4 | 11 | −25 to −44 | 7.10E+08 | This study |
| Kagoshima Bay (Wakamiko crater) | plume | 7 | 40 | 1 to −30 | 9.30E+08 | This study |
| Rivalta (Italy) | MV |  | 793 | −41.4 |  | [1] |
| Pineto (Italy) | MV |  | 694 | −73.1 |  | [1] |
| Bazna (Romania) | MV | 0.02 | 39.64 |  |  | [2] |
| Paclele Mari (Romania) | MV | 0.04 | 89.38 | −37.9 |  | [2-3] |
| Wushanding (Taiwan) | MV |  | 3170 | −30.3 |  | [4] |
| Luoshan (Taiwan) | MV | 1.9 | 190 | −38.8 | 1.24E+09 | [4-7] |
| Chunglun (Taiwan) | MV | 5.9 | 4760 | −38.4 | 6.05E+09 | [4-8] |
| Chinshui (Taiwan) | gas well | 1.8 | 0.07 |  | 1.08E+10 | [5,9] |
| Chuhuangkeng (Taiwan) | gas well | 3.6 | 0.02 |  | 1.97E+09 | [5,9] |
| Gas Hydrate Potential Area (offshore SW Taiwan) | plume | 0.2-0.4 | 0.1 | -74.6 | 4.88E+08 | [10-11] |

**References**

[1] Etiope, G., Martinelli, G., Caracausi, A. & Italiano, F. Methane seeps and mud volcanoes in Italy: Gas origin, fractionation and emission to the atmosphere. *Geophys. Res. Lett.* **34** (2007).

[2] Baciu, C., Caracausi, A., Etiope, G. and Italiano, F. Mud volcanoes and methane seeps in Romania: main features and gas flux. Ann. Geophys. 50, 501-511 (2007).

[3] Etiope, G., Baciu, C., Caracausi, A., Italiano, F. and Cosma, C. Gas flux to the atmosphere from mud volcanoes in Eastern Romania. Terra Nova, 16, 179-184 (2004).

[4] Yang, T. F., Chen, C. H., Tien, R. L., Song, S. R. & Liu, T. K. Remnant magmatic activity in the Coastal Range of East Taiwan after arc-continent collision: fission-track data and ^3^He/^4^He ratio evidence. Radiat Meas **36**, 343-349 (2003).

[5] Yang, T. F. et al. Composition and exhalation flux of gases from mud volcanoes in Taiwan. Environ Geol 46, 1003-1011 (2004).

[6] You, C. F., Gieskes, J. M., Lee, T., Yui, T. F. & Chen, H. W. Geochemistry of mud volcano fluids in the Taiwan accretionary prism. *Appl Geochem* **19**, 695-707 (2004).

[7] Chao, H.C., You, C.F. and Sun, C.H. Gases in Taiwan mud volcanoes: Chemical composition, methane carbon isotopes, and gas fluxes. Appl. Geochem. **25**, 428-436 (2010).

[8] Yang, T. F. Recent progress in the application of gas geochemistry: examples from Taiwan and the 9th International Gas Geochemistry Conference. *Geofluids* **8**, 219-229 (2008).

[9] Sano, Y., Wakita, H. & Huang, C. W. Helium flux in a continental land area estimated from 3He/4He ratio in Northern Taiwan. Nature **323**, 55-57 (1986).

[10] Chuang, P. C. et al. Estimation of methane flux offshore SW Taiwan and the influence of tectonics on gas hydrate accumulation. Geofluids 10, 497-510 (2010).

[11] Yang, T. F. *et al.* Methane venting in gas hydrate potential area offshore of SW Taiwan: Evidence of gas analysis of water column samples. *Terr Atmos Ocean Sci* **17**, 933-950 (2006).
